# Medicaid waiver-based housing supports in a safety-net setting: utilization and spending outcomes

**DOI:** 10.1093/haschl/qxag244

**Published:** 2026-09-04

**Authors:** Nicole C McCann, Heather E Hsu, Stephanie Ettinger de Cuba, Jasper Frank, Paulina Lange, Michael D Stein, Paul R Shafer

**Affiliations:** Department of Health Law, Policy, and Management, Boston University School of Public Health, Boston, MA 02118, United States; Department of Pediatrics, Boston Medical Center, Boston, MA 02118, United States; Department of Pediatrics, Boston University Chobanian and Avedisian School of Medicine, Boston, MA 02118, United States; Department of Health Law, Policy, and Management, Boston University School of Public Health, Boston, MA 02118, United States; Department of Pediatrics, Boston University Chobanian and Avedisian School of Medicine, Boston, MA 02118, United States; Department of Population Health, Boston Medical Center, Boston, MA 02118, United States; Department of Population Health, Boston Medical Center, Boston, MA 02118, United States; Department of Health Law, Policy, and Management, Boston University School of Public Health, Boston, MA 02118, United States; Department of Health Law, Policy, and Management, Boston University School of Public Health, Boston, MA 02118, United States

**Keywords:** Medicaid, Section 1115 demonstration waivers, health-related social needs, housing instability, tenancy supports

## Abstract

**Introduction:**

States have expanded Medicaid waiver-based housing support programs, but evidence on outcomes in safety-net settings is limited.

**Methods:**

We evaluated health care utilization and spending associated with Massachusetts Medicaid's Flexible Services Program housing supports (FSP-H), provided to Accountable Care Organization (ACO) members with housing-related needs and qualifying health risks (2021-2024). Within a Boston-based safety-net health system, we linked FSP-H data to claims and electronic health records. Using staggered difference-in-differences, we compared monthly probability of inpatient and emergency department (ED) utilization and per-patient spending in FSP-H recipients (*n* = 340) vs a contemporaneous comparison group with documented housing-related needs who were FSP-H-ineligible because they were in non-ACO plans (*n* = 425). We conducted clinical risk-based subgroup analyses.

**Results:**

Overall, FSP-H receipt was not associated with significant changes in monthly probability of inpatient utilization (−3.3 percentage points, *P* = 0.223), ED utilization (−1.5 percentage points, *P* = 0.569), or monthly spending (−$634, *P* = 0.053). Massachusetts Medicaid's Flexible Services Program housing supports (FSP-H) was significantly associated with lower monthly inpatient utilization in the substance use disorder and frailty subgroups and with lower monthly spending in the frailty subgroup.

**Conclusion:**

Findings offer evidence for state Medicaid plans implementing or adapting housing programs.

## Introduction

Housing is a critical health-related social need (HRSN); homelessness and housing instability are associated with adverse health outcomes, mortality, and costly health care utilization.^1-7^ In 2024, an estimated 771 480 people in the United States were homeless on a given night, an 18% increase from 2023.^8^ Housing instability is also widespread: in 2023, 17% of households spent over half their income on housing or lived in severely inadequate conditions.^9^

Given links between housing, health, and costs, health care organizations, including payors, have invested in housing programs. The Centers for Medicare and Medicaid Services (CMS) previously expanded these efforts through Medicaid Section 1115 waiver opportunities, which allow states to test strategies to improve health outcomes.^10,11^ Under the Biden Administration, addressing HRSN became a priority, with 25 states securing HRSN-focused waivers, most including housing supports.^12^

In 2020, Massachusetts Medicaid (MassHealth) used the authority granted in a 2017-2022 section 1115 waiver extension to launch the Flexible Services Program (FSP), which leverages state Medicaid funding to address HRSN for eligible Accountable Care Organization (ACO) members. Since then, FSP was rolled out across the state's 17 ACOs, with a goal to improve health outcomes and reduce costly health care utilization for members with nutrition or housing-related needs.^13^ The housing supports component of FSP (MassHealth Flexible Services Program housing supports [FSP-H]) involved contracting between the ACOs and social service organizations to provide pretenancy (eg, housing application assistance), transitional items (eg, furniture), tenancy-sustaining (eg, tenancy training), or home modification (eg, grab bar installation) supports.^14^ To be eligible, members had to have a housing-related need (homelessness, housing instability) and a health need indicating clinical risk, including complex physical or mental health conditions, or substance use disorders (SUD).^14^

Housing support interventions can improve health outcomes and reduce costs,^15,16^ but program designs vary, limiting generalizable conclusions.^17,18^ Evidence on housing supports delivered through Medicaid-based programs is mixed, and success may depend on implementation strategies.^19-21^ Prior statewide evaluations of MassHealth FSP found reduced acute health care utilization associated with nutrition supports overall and with housing supports among individuals with behavioral health conditions.^22,23^ Expanding the evidence base on Medicaid-based housing supports, including analyses in local settings and across varied populations, is essential to inform future program and policy decisions, particularly given the current Administration's withdrawal of federal guidance supporting HRSN initiatives.^24^ Safety-net health systems are especially important settings for evaluating such initiatives, as they care for patients with substantial HRSNs, complex health conditions, and barriers to health care and social support.

We described service delivery and evaluated health care utilization and spending outcomes associated with MassHealth FSP-H. To our knowledge, this is the first study to evaluate MassHealth FSP housing supports in a safety-net setting and to explore variation across patients with different health conditions and clinical risk.

## Methods

### Setting and data

We evaluated FSP-H implementation within a single Boston-based ACO governed by a partnership between WellSense Health Plan, a managed care organization, and the Boston Medical Center Health System (BMCHS), the largest safety-net health care system in New England (Supplemental Methods).^25,26^ Approximately a quarter of hospitalized BMCHS patients experience homelessness, and a third have SUD, making this a high-need population for social support interventions.^27,28^

To conduct our retrospective cohort study, we linked FSP-H programmatic records, WellSense Plan data, and electronic health record (EHR) data (Supplemental Methods). Programmatic data included FSP-H enrollment and service delivery information. WellSense Plan data included claims for MassHealth and qualified health plan enrollees (both ACO and non-ACO plans), including plan enrollment, demographics, utilization, and spending files.

### Sample

Programmatic FSP-H eligibility criteria included experiencing or being at risk of homelessness and having a health need based on MassHealth-provided definitions (Table S1).^14^ We identified FSP-H recipients who received ≥1 FSP-H service during the program data collection period (March 2021 to November 2024). The exposure therefore represents receipt of the housing support intervention, whether or not the supports resulted in stable housing.

In weighted and covariate-adjusted comparative analyses of utilization and spending outcomes, we included FSP-H recipients who were continuously enrolled in their ACO plan for at least 3 months before and 3 months after the month of their first FSP-H service to reduce confounding from plan switching. To identify the comparison group, we included individuals who had housing-related needs but were ineligible for FSP-H because they were not in an ACO plan. This ineligibility reduced bias from time-varying unobserved factors that influence FSP-H participation. To construct this group, we identified BMCHS patients enrolled in a WellSense-managed non-ACO MassHealth plan (ie, Managed Care Organization or qualified health plan) for at least 7 months (mirroring the FSP-H exposed group minimum study period) (Supplemental Methods). We used a validated EHR-based algorithm, which aggregates social needs screening results, housing consult orders, diagnostic codes, registration indicators, and address information, to restrict the comparison group to individuals with an identified prior-year housing-related need in each month of their study eligibility span between 2021 and 2023.^29^ Electronic health record algorithm data from 2024 were not available due to HRSN screening process changes (Supplemental Methods). For both groups, individuals were excluded if they enrolled in another housing program during this period (ie, the Living Well at Home program administered by BMCHS).

### Outcomes

Primary outcomes were monthly probability of (1) inpatient and (2) emergency department (ED) utilization. These were identified using encounter-level claims and defined as binary indicators equal to 1 if an individual had any utilization during a given month, and 0 otherwise (Supplemental Methods). A secondary outcome was monthly per-patient spending defined as the total dollar amount paid by the insurance plan to providers per individual per month (ie, billed services). MassHealth Flexible Services Program housing supports services themselves were not billable, so were not included in the spending outcomes. To adjust for outliers in difference-in-differences regression, monthly spending was top-coded at $32,000, our sample's 99th percentile.^30^ Spending data were not available for 2024 (Supplemental Methods).

### Covariates

Demographic covariates included age, gender, race and ethnicity, and preferred language. Demographic variables were based on claims documentation at FSP-H start (FSP-H recipients) or first included month (comparison group). For race/ethnicity and language, which had high missingness, we created missing categories to preserve sample size and statistical power.

Clinical covariates were based on presence of ≥1 International Classification of Diseases 10th Edition (ICD-10) diagnosis, Healthcare Common Procedure Coding System code, or Current Procedural Terminology code. We used modified Elixhauser score, which aggregates comorbid conditions, excluding separately reported behavioral conditions (Table S2).^31^ Categories were 0, 1, 2, or 3+ conditions. Other clinical characteristics were selected based on clinical risk-based FSP-H inclusion criteria^32^ and defined by presence of ≥1 code in the 13 months before and including the month of FSP-H start (FSP-H recipients) or study inclusion (comparison group). Conditions included SUD, serious mental illness (SMI), and frailty associated with dependence for activities of daily living (ADLs) (Table S2).^31,33^

### Analyses

#### Service delivery

We reported the number of FSP-H recipients, the proportion experiencing homelessness vs housing instability at program start, the number of days between individuals' first and last service receipt, and the number of services per individual. We also reported the number of services delivered to all recipients, and the proportion of each service type (Table S3).

#### Utilization and spending

We described FSP-H and comparison group demographic and clinical characteristics and tested for differences between groups. For FSP-H recipients, we reported unadjusted monthly outcomes over a 12-month preperiod before FSP-H start and a 6-month postperiod follow-up.

We used difference-in-differences to evaluate change in outcomes before and after FSP-H receipt compared with unexposed comparators. To account for treatment timing variation, we used a staggered difference-in-differences approach developed by Callaway and Sant’Anna, which avoids pitfalls of standard 2-way fixed effects models when treatment timing varies while still adjusting for secular trends.^34^ This approach groups treated units by treatment timing and compares them only to valid contemporaneous controls, including never-treated and not-yet-treated FSP-H (Supplemental Methods). We implemented the default specification, which combines inverse-probability-of-tilting weights with weighted least squares regression adjustment.^35,36^ With this doubly robust approach, the weighting first balances observed covariates between groups, and regression adjustment accounts for residual observed confounding, meaning the estimator remains consistent if either the weighting model or the regression model is correctly specified.

For valid difference-in-differences interpretation, preperiod outcome trends must be parallel. We assessed parallel trends with an event study specification and considered this assumption met if the FSP-H and comparison groups showed no significant differences in monthly or overall trends during the 12-month preperiod.

Our primary effect estimate was the simple average treatment effect on the treated (ATT), which weights group-level ATTs to produce an aggregate coefficient (Supplemental Methods).^37^ We also reported monthly event study estimates for the postperiod to assess dynamic outcome trends.

We conducted subgroup analysis among individuals with the clinical conditions specified above. We also evaluated the ATT in individuals above and below age 55. Subgroups aligned with evolving eligibility criteria thresholds.^32^

### Sensitivity analyses

For overall comparative analyses, we additionally evaluated dynamic and aggregate effect estimates using an unadjusted, ordinary least squares (OLS) regression to assess pre- and posttrends unconditional on covariates. For inpatient and ED utilization, we analyzed encounter counts, rather than binary probabilities. For monthly spending, we conducted analyses without top-coding.

We used STATA Version 17.0 for analysis (conducted January December 2025). We followed Strengthening the Reporting of Observational Studies in Epidemiology (STROBE) reporting guidelines. We applied a prespecified threshold of *P* < 0.05 to determine statistical significance.

## Results

### Service delivery

Overall, 437 individuals received ≥1 FSP-H service, with 68% experiencing homelessness and 32% experiencing housing instability. Individuals received a median of 2 services over a median of 62 days. In total, 1148 services were documented between March 2021 and November 2024. Pretenancy ongoing coordination was most frequent (40%), followed by tenancy-sustaining ongoing coordination (27%), and pretenancy transitional fees and payments (12%) (Table S4).

### Utilization and spending: analytic sample

Our comparative analysis sample included 340 FSP-H recipients and 425 non-ACO comparators who met inclusion criteria. The FSP-H and comparison groups were significantly different across all reported demographic and clinical characteristics except gender (Table 1).

**Table 1.** Comparative analytic sample: demographic and clinical characteristics FSP-H recipients and ineligible comparison group, 2021-2024.

Characteristic^a^	Ineligible comparison group (*N* = 425)	FSP-H recipients (*N* = 340)	*P-*value^b^
Age category, years, n (%)			
17-30	46 (11)	38 (11)	0.006
31-40	126 (30)	69 (20)	
40-54	143 (34)	112 (33)	
55+	110 (26)	121 (36)	
Gender,^c^ n (%)			
Female	215 (51)	168 (49)	0.746
Male	210 (49)	172 (51)	
Race/ethnicity^c^, n (%)			
Non-Hispanic white	56 (13)	65 (19)	0.001
Non-Hispanic Black	119 (28)	88 (26)	
Hispanic	49 (12)	19 (6)	
Asian^d^	<11 (<3)	<11 (<3)	
Hawaiian or Pacific Islander^d^	<11 (<3)	<11 (<3)	
American Indian or Alaska Native^d^	<11 (<3)	<11 (<3)	
Missing	194 (46)	161 (47)	
Language, n (%)			
English	178 (42)	168 (49)	<0.001
Spanish	42 (10)	12 (4)	
Other	27 (6)	12 (4)	
Missing	178 (42)	148 (44)	
Modified Elixhauser score, n (%)			
0 conditions	121 (28)	45 (13)	<0.001
1 condition	114 (27)	61 (18)	
2 conditions	74 (17)	53 (16)	
3+ conditions	116 (27)	181 (53)	
Substance use disorder^e^, n (%)	122 (29)	172 (51)	<0.001
Serious mental illness^e^, n (%)	133 (31)	197 (58)	<0.001
Frailty associated with ADL dependence, n (%)^f^	251 (59)	268 (79)	<0.001

Data derived from authors' own analyses of FSP-H, claims, and electronic health records from WellSense and Boston Medical Center Health System.

Abbreviations: ADLs, activities of daily living; FSP-H, MassHealth Flexible Services Program housing supports.

^a^Characteristics at FSP-H initiation (for FSP-H recipients), or first month of study inclusion (for comparison group).

^b^Chi-square tests were used to calculate *P-*values; Fisher's exact test was applied with expected cell counts <5. For variables with missing categories, *P*-values were calculated excluding missing responses.

^c^Gender and race/ethnicity as documented in claims data.

^d^Asian, Hawaiian or Pacific Islander, and American Indian or Alaska Native, race/ethnicity categories aggregated have cell counts <11; Ns redacted per MassHealth guidelines.

^e^As indicated by ≥1 ICD-10 or procedure code in the 13-month period leading up to and including program engagement (for FSP-H recipients) or first month of study inclusion (for comparison group).

^f^ Table S2 details all diagnostic and procedure codes used to define frailty, based on a previously published frailty index.

### Utilization and spending: FSP-H recipient pre- and postoutcomes

In FSP-H recipients, the unadjusted average monthly inpatient utilization rate (ie, proportion with an inpatient visit) was 14.7% (95% CI: 13.7%, 15.7%) during the preperiod and 13.6% (95% CI: 12.1%, 15.1%) in the postperiod. The mean monthly ED utilization rate was 15.8% (95% CI: 14.6%, 17.1%) in the preperiod and 14.5% (95% CI: 13.3%, 15.7%) in the postperiod. Mean monthly per-patient spending was $3803 (95% CI: $3,534, $4071) in the preperiod and $3166 (95% CI: $2,760, $3571) in the postperiod. Month-by-month values for these outcomes and median spending are in Table S5.

### Comparative analysis: monthly utilization

**Figure 1.**
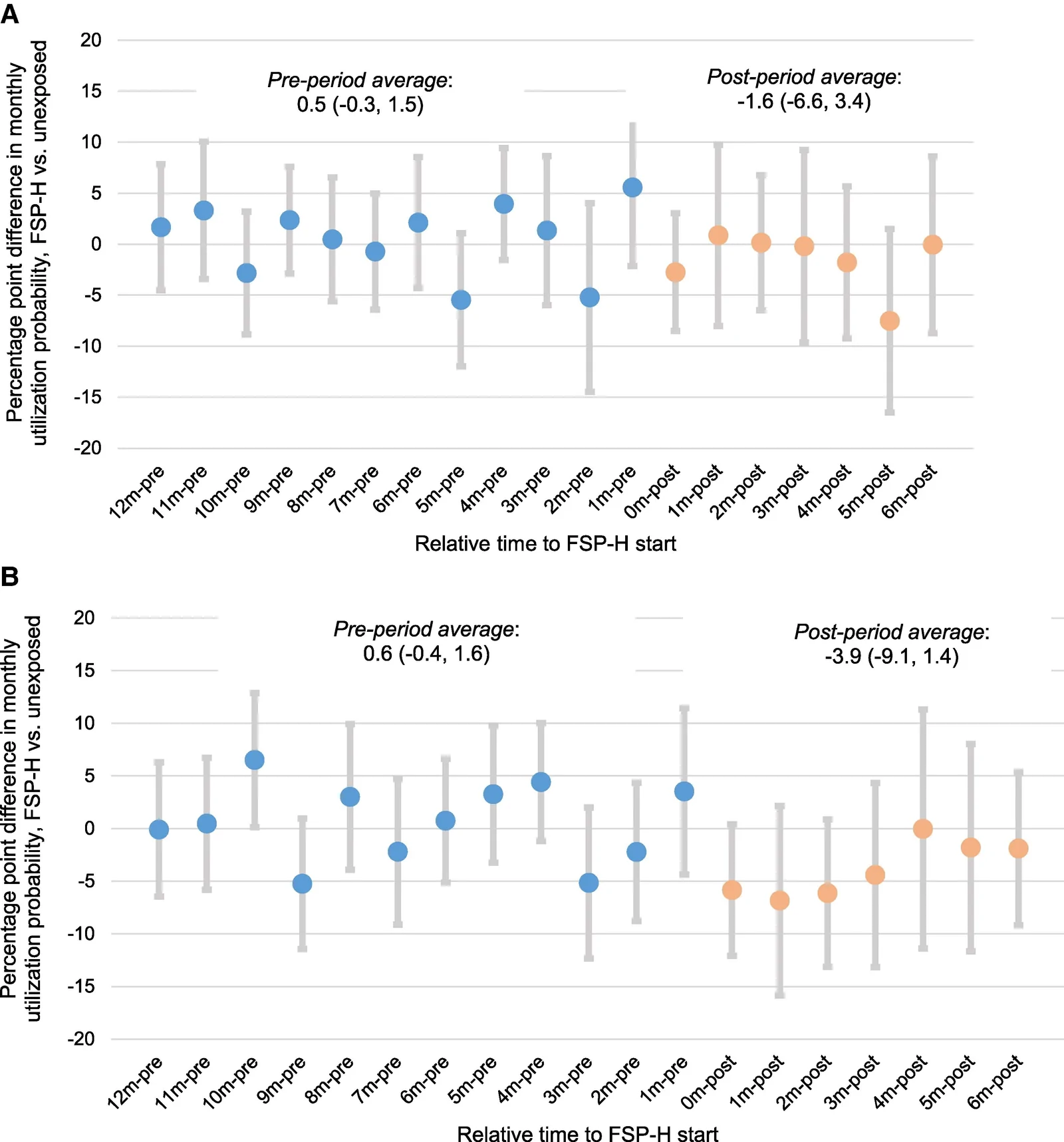
Difference-in-differences event study plot: utilization outcomes in FSP-H vs unexposed comparators, 2021-2024. Data derived from authors' own analyses of FSP-H, claims, and electronic health records from WellSense and Boston Medical Center Health System. Difference-in-differences event study plots for inpatient (A) and emergency department (B) utilization. Plots show the ATT between FSP-H and unexposed comparison groups, in each month relative to FSP-H start, from 12 months prior (ie, 12m-pre) to 6 months after (ie, 6m-post). Preperiod and postperiod averages are shown. The doubly robust model adjusted for demographics and clinical characteristics using inverse probability of tilting weights and regression adjusted via weighted least squares. Note that pre- and postaverages differ from the aggregate ATT (described in the text and Table 2), as they are simple averages across time and not weighted by group size. Abbreviation: ATT, average treatment effect on the treated; FSP-H, MassHealth Flexible Services Program housing supports.

Our event study showed that 12-month preperiod differences in inpatient and ED utilization between FSP-H recipients and unexposed comparators had confidence intervals centered around zero, supporting the parallel trends assumption (Figure 1). In the 6-month postperiod, differences in inpatient and ED utilization were not significantly different between FSP-H and unexposed groups. However, results indicated a trend toward decreased utilization in FSP-H recipients, with confidence intervals shifting negative (Figure 1). The aggregate ATTs for inpatient and ED utilization were not statistically significant but suggested potential decreased utilization in FSP-H recipients (Table 2), with an overall 3.3 percentage point decrease in inpatient utilization (95% CI: −8.2, 2.0, *P* = 0.223) and a 1.5 percentage point decrease in ED utilization (95% CI: −6.5, 3.6, *P* = 0.569).

**Table 2.** Changes in utilization and spending outcomes associated with FSP-H compared with unexposed comparators, overall and by subgroup, 2021-2024.

Average treatment effect on the treated (ATT)	Inpatient utilization, percentage point change in monthly probability		ED utilization, percentage point change in monthly probability		Change in per-patient monthly spending^a,b^, US $	
	Coefficient (95% CI)	*P*-value	Coefficient (95% CI)	*P*-value	Coefficient (95% CI)	*P*-value
Overall	−3.3 (−8.7, 2.0)	0.223	−1.5 (−6,5, 3.6)	0.569	−634 (−1,277, 9)	0.053
3+ modified Elixhauser conditions	−2.9 (−14.3, 8.5)	0.621	−2.1 (−11.1, 6.9)	0.644	−171 (−2,091, 1748)	0.861
Substance use disorder	−13.6 (−25.1, −2.0)	0.021	−5.8 (−17.9, 6.4)	0.352	−962 (−2,229, 305)	0.137
Serious mental illness^c^	−6.7 (−15.3, 1.9)	0.126	−7.1 (−16.3, 2.1)	0.128	−1211 (−2,187, −235)	0.015
Frailty associated with ADL dependence	−7.2 (−13.5, −0.8)	0.027	−4.0 (−10.2, 2.3)	0.213	−851 (−1,658, −43)	0.039
Age 17-54	−3.6 (−10.5, 3.3)	0.303	−0.9 (−7.1, 5.2)	0.767	−378 (−1,136, 379)	0.327
Age 55+	−1.8 (−11.6, 8.0)	0.714	−3.5 (−13.6, 6.5)	0.491	−1326 (−2,732, 81)	0.065

Data derived from authors' own analyses of FSP-H, claims, and electronic health records from WellSense and Boston Medical Center Health System.

Abbreviations: ADLs, activities of daily living; ATT, average treatment effect on the treated; ED, emergency department; FSP-H: MassHealth Flexible Services Program housing supports.

^a^Spending was top-coded at the 99th percentile in the study population to account for outliers; non-top-coded results are shown in Table S7.

^b^Spending data were not available for 2024 (Supplemental Methods).

^c^The parallel trends assumption did not hold for the subgroup of individuals with serious mental illness for the monthly spending outcome, therefore, this association should be interpreted with caution.

In subgroup analyses, the parallel trends assumption consistently held (Table S6). In all subgroups, ATT coefficients were negative, with most nonsignificant. However, among individuals with SUD and frailty associated with ADL dependence, decreases reached statistical significance for inpatient utilization (Table 2). Among individuals with SUD, FSP-H was associated with a 13.6 percentage point decrease in inpatient utilization (95% CI: −25.1, −2.0; *P* = 0.021), and among individuals with frailty associated with ADL dependence, FSP-H was associated with a 7.2 percentage point decrease in inpatient utilization (95% CI: −13.5, −0.8; *P* = 0.027).

Results regarding an overall nonsignificant, negative shift in utilization did not substantially vary when we used unadjusted OLS models or when evaluating inpatient and ED monthly counts (Table S7; Figure S1).

### Comparative analysis: monthly spending

**Figure 2.**
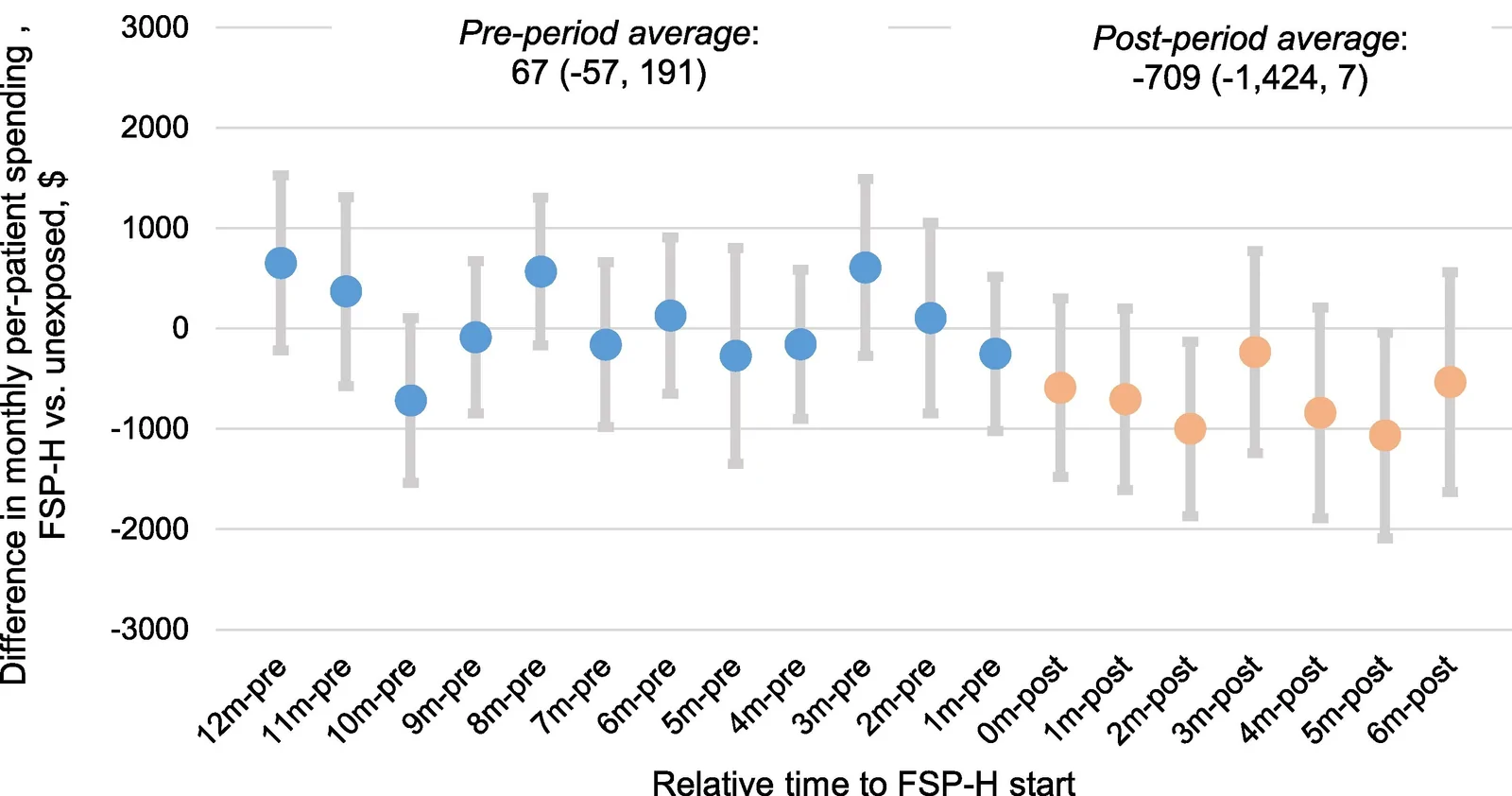
Difference-in-differences event study plot: per-patient monthly spending in FSP-H vs unexposed comparators, 2021-2024. Data derived from authors' own analyses of FSP-H, claims, and electronic health records from WellSense and Boston Medical Center Health System. Difference-in-differences event study plot for per-patient monthly spending. Monthly spending is capped at the sample's 99th percentile. Shows the ATT between FSP-H and unexposed comparison groups, in each month relative to FSP-H start, from 12 months prior (ie, 12m-pre) to 6 months after (ie, 6m-post). Preperiod and postperiod averages are shown. The doubly robust model adjusted for demographics and clinical characteristics using inverse probability of tilting weights and regression adjusted via weighted least squares. Note that pre- and postaverages differ from the aggregate ATT (described in the text and Table 2), as they are simple averages across time and not weighted by group size. Abbreviations: FSP-H, MassHealth Flexible Services Program housing supports; ATT, average treatment effect on the treated.

In the event study, preperiod differences in monthly per-patient spending between FSP-H recipients and unexposed comparators were stable, supporting the parallel trends assumption (Figure 2). In the postperiod, FSP-H recipient spending decreased relative to comparators; however, decreases were not statistically significant in most months (Figure 2). Overall, the aggregate ATT suggested a $634 decrease (95% CI: −$1,277, $9, *P* = 0.053) in monthly per-patient spending associated with FSP-H receipt (Table 2), approaching statistical significance.

In subgroup analyses, the parallel trends assumption held, except in the SMI subgroup, in which the preperiod average event study coefficient was statistically significant; thus, results for this group cannot be confidently interpreted (Table S6). Overall, monthly per-patient spending decreased in all subgroups, with a statistically significant difference observed in those with frailty associated with ADL dependence (Table 2).

In sensitivity analysis, preperiod, postperiod, and aggregate results were similar using an unadjusted OLS model (Table S7; Figure S2). Without top-coding, the spending coefficient magnitude was similar, with a larger *P*-value (Table S7).

## Discussion

Within this safety-net Boston ACO, 437 individuals received FSP-H between March 2021 and November 2024. The program most commonly provided pretenancy coordination (40%) and tenancy-sustaining coordination (27%), followed by household items (15%) and fees and payments for those transitioning into housing (12%). In difference-in-differences analysis, we did not find significant decreases in monthly inpatient and ED utilization associated with FSP-H receipt overall. However, there were significant declines in inpatient utilization among individuals with SUD and frailty diagnoses. Monthly per-patient spending did not decrease using our prespecified significance threshold of *P* = 0.05; however, trends pointed toward a decrease (−$634, *P* = 0.053), with significant reductions among individuals with frailty diagnoses. Despite sample size limitations, findings may provide suggestive evidence for MassHealth and other states considering similar programs, while also highlighting the need for further evaluation.

Findings add to emerging evidence showing mixed results regarding outcomes of Medicaid waiver-based housing support programs. A large statewide study of FSP-H across all Massachusetts ACOs reported lower health care utilization among individuals with behavioral health conditions vs 2 comparison groups (program-eligible but unenrolled and program-ineligible).^22^ An evaluation of North Carolina's Healthy Opportunities Pilot, which included housing and/or nutrition supports, reported decreased health care spending among recipients.^20^ Another study of FSP-H in a higher-resourced single ACO setting evaluated changes in food and housing insecurity, as well as health care utilization outcomes in FSP recipients, finding no differences vs an eligible, unenrolled comparison group. However, it only included 16 individuals receiving housing supports.^38^ Notably, a study that evaluated several California Medicaid-based HRSN pilot programs found substantial variation in outcomes, some reducing utilization and some having no impact, suggesting that setting-specific variation in health system characteristics, program design, implementation, and included populations contribute to heterogeneous findings.^19^

Our study is the first to our knowledge to evaluate health care utilization and spending associated with MassHealth FSP-H in a safety-net setting. Evaluating housing supports is challenging, limited by small samples, short follow-up time, and low-quality data on housing need resolution. Still, our study adds to the emerging body of evidence and contributes to the understanding of MassHealth FSP-H in a lower-resourced safety-net setting population with high social and clinical need and with varied clinical risk characteristics. We highlighted substantial housing support delivery, an important process outcome, and a potential association between FSP-H and reduced utilization and spending among individuals with certain clinical diagnoses, informing future program evaluation and improvement efforts.

Our analysis was conducted in a safety-net setting serving patients with HRSNs and complex clinical conditions; over 50% of FSP-H participants had SUD, SMI, or frailty diagnoses. Our results suggest that outcomes may vary by clinical characteristics and risk. For example, a larger association between program receipt and reduced inpatient utilization was observed among FSP-H recipients with SUD and frailty diagnoses than those without. More research is needed to understand mechanisms driving this, and whether they reflect differences in program implementation or effectiveness. Further, our subgroup analyses may be useful for understanding recent changes to MassHealth FSP and could be informative for future decision-making. In 2025, MassHealth FSP transitioned to an ACO-covered Managed Care-based HRSN Supplemental Services program. Simultaneously, MassHealth restricted eligibility criteria in response to CMS funding constraints and guidance that shifted all financial risk to the state. We projected that almost 70% of FSP-H recipients from 2021 to 2024 would no longer be eligible under the new criteria.^32^ Restrictions included an age limit for pretenancy services to adults who are 55+. Our findings suggest that clinical conditions may be stronger indicators of decreased acute care utilization after FSP-H enrollment than age. These findings are also consistent with the statewide evaluation reporting decreased utilization and spending among individuals with behavioral health conditions.^22^

Our study has strengths and limitations. First, to minimize selection bias related to program engagement, we constructed a comparison group with documented housing-related needs that was ineligible for FSP-H due to being in a non-ACO plan. Accountable Care Organization vs other Medicaid managed care plan enrollment is primarily based on patient primary care providers' choice to join the ACO (a quasi-random factor) and, to a lesser degree, patient personal choice (nonrandom), while ACO vs qualified health plan enrollment is based on Medicaid eligibility (ie, income). With this, there were significant baseline differences between FSP-H recipients and non-ACO comparators. To mitigate this, the difference-in-difference approach was supported by the parallel trends assumption, which posits that even if group differences exist, the differences are accounted for as long as they are constant over time (eg, sociodemographics). Further, the staggered approach enabled not-yet-treated individuals (those using FSP-H in the future) to contribute to comparisons with treated individuals, resulting in smaller differences in group characteristics than if only never-treated units (not eligible for FSP-H) were included. Despite this quasi-experimental design, we could not adjust for unobserved, time-varying factors associated with both FSP-H receipt and outcomes (eg, help seeking) and therefore described results conservatively as associations. In particular, regression to the mean is possible and could bias results toward significance. However, our descriptive results for outcomes in the overall FSP-H group prior to treatment do not indicate a triggering event immediately before FSP-H initiation, as utilization and spending fall within the range of the full preperiod (Table S5), and the overall analyses were insignificant, partially mitigating this concern. Subgroup analyses are more vulnerable to bias because of smaller sample sizes and wider confidence intervals around the parallel trends estimates. However, in subgroups with significant outcomes, differences in outcomes relative to the comparison group in the month immediately preceding FSP-H initiation were either negative (for those with substance use disorder and inpatient utilization and those with frailty and spending), or relatively small (for those with frailty and inpatient utilization) (Table S6), providing little evidence of a pronounced pretreatment triggering event that would be expected if regression to the mean were driving the observed effects. Relatedly, with our small sample size and wide confidence intervals, particularly in monthly and subgroup analyses, we de-emphasize binary conclusions regarding statistical significance and instead highlight overarching outcome trends and acknowledge uncertainty. Third, follow-up survey data on housing-related need resolution were limited in our sample, and we could not differentiate between housing support receipt and housing problem resolution, making it difficult to interpret outcomes as the result of implementation factors or the program. There was also significant heterogeneity within the FSP-H services, and we did not differentiate outcomes by service type or social service organization. We may underestimate the impact of gaining stable housing, as this analysis evaluates FSP-H as a package of varied housing support services rather than housing itself. However, this is informative from a health system resource utilization standpoint. Last, we only evaluated utilization and spending, as the ACO's goal was to reduce these. FSP-H could have other unmeasured impacts which should be studied in future work (eg, quality of life).

In conclusion, findings in our relatively small sample of 340 FSP-H recipients in a safety-net setting were not significant overall but suggest potential reductions in health care utilization and/or spending associated with FSP-H participation among individuals with high clinical risk, including SUD and frailty diagnoses. Null overall findings may reflect challenges of health system-based housing supports, which are inherently limited by housing market factors including supply and affordability. Nevertheless, health systems are important touch points for connecting patients with social services. Evaluation of housing supports provided through health systems remains in early stages, following the rapid expansion of Medicaid waiver-based HRSN programs under the Biden Administration. Medicaid waivers are intended to build an evidence base to inform states' decision-making on program adoption, implementation, and improvement. We add to the emerging mixed evidence on Medicaid-based housing support programs and emphasize the importance of sustained investment in evaluation to better understand programs' impacts on individuals and health systems, and to inform continuous improvements.

## Supplementary Material

qxag244_Supplementary_Data
