# Supplementary material for: Medicaid waiver-based housing supports in a safety-net setting: utilization and spending outcomes
Source: Health Aff Sch. 2026 Sep 4;4(9):qxag244. doi: 10.1093/haschl/qxag244 (PMC13587248; doi:10.1093/haschl/qxag244)
Supplement: Supplementary file 1 [file qxag244_supplementary_data.zip › Supplemental material.docx]

**Supplemental Appendix:**

**Medicaid Waiver-Based Housing Supports in a Safety-Net Setting: Utilization and Spending Outcomes**

**Table of contents.**

|  | *Page* |
| --- | --- |
| **Supplemental Methods.** Setting and data, sample, outcomes, and analyses | 2-4 |
| **Supplemental Table 1.** Summary of MassHealth eligibility criteria for FSP-H | 5 |
| **Supplemental Table 2**. ICD-10 and procedure codes used to identify clinical covariates | 6-8 |
| **Supplemental Table 3.** Housing support service types available in FSP-H | 9 |
| **Supplemental Table 4.** FSP-H recipients and service delivery, 2021-2024 | 10 |
| **Supplemental Table 5**. Mean monthly inpatient and ED utilization, and per-patient spending in FSP-H recipients before and after FSP-H, 2021-2024 | 11 |
| **Supplemental Table 6.** Pre-period trends by subgroup: monthly inpatient utilization, emergency department utilization, and per-patient spending, 2021-2024 | 12-14 |
| **Supplemental Table 7.** Staggered difference-in-difference sensitivity analyses | 15 |
| **Supplemental Figure 1**. Difference-in-differences event study plot, unadjusted ordinary least squares (OLS) model: utilization outcomes in FSP-H vs. unexposed comparators, 2021-2024 | 16 |
| **Supplemental Figure 2:** Difference-in-differences event study plot, unadjusted ordinary least squares (OLS) model: per-patient monthly spending in FSP-H vs. unexposed comparators, 2021-2024 | 17 |
| **Supplemental Endnotes** | 18 |
|  |  |
|  |  |

**Supplemental methods**

*Setting and data*

WellSense Health Plan is a managed care organization offering MassHealth and qualified commercial insurance plans. The Boston Medical Center Health System includes Boston Medical Center and affiliated community health centers. The WellSense Community Alliance Accountable Care Organization involves a partnership between WellSense Health Plan and BMCHS in which these organizations govern ACO-based programs run through MassHealth contracts, including MassHealth FSP.^1^

We linked internal BMCHS programmatic data, claims, and electronic health record (EHR) data. BMCHS programmatic data contained FSP-H patient identifiers (linkable to claims and EHR), program enrollment records, patients’ documented housing-related need at enrollment (homelessness or housing instability), service delivery dates, service category (pre-tenancy, tenancy-sustaining, or home modification), and specific services delivered (e.g., ongoing coordination, fees and payments, household items). We also used WellSense claims data, which contained statewide claims data for enrollees in MassHealth plans administered by WellSense. This dataset included a demographic file, an enrollment file with information on insurance plan type and enrollment dates, encounter-level files with encounter types, diagnostic and procedure code files, and spending files. Our third data source was the BMCHS electronic health record (EHR), which contained documentation for BMCHS patients, including the subset of WellSense members who received care at BMC. The BMCHS EHR included housing-related information, which could be queried using a validated structured data element-based algorithm to identify patients with homelessness or housing instability.^2^

*Sample*

We included a minimum study period of seven months for both FSP-H recipients and comparison group members. This minimum period was selected based on prior literature which has evaluated utilization outcomes of HRSN interventions over a 3-month follow-up period,^3^ and we opted to include at least the same amount of time in the pre-period. However, individuals could contribute additional person-month data as long as they continuously met inclusion criteria (i.e., continuous enrollment and housing-related need criteria) prior to and after this minimum study period. The staggered difference-in-differences approach allows for this flexibility for individuals to contribute varying amounts of panel data, as two-way comparisons are conducted in calendar time.

The staggered difference-in-differences approach also provides flexibility in cases with unbalanced panel data. In this study, we had an unbalanced panel because FSP-H recipients could receive treatment between 2021-2024, and comparison group data were only available for 2021-2023. The analytic approach accounts for this by only conducting valid comparisons in calendar time. A limitation is that units without contemporaneous comparisons are dropped from analysis, restricting the sample size in calendar time periods with no overlapping treated and unexposed comparators. However, individuals treated in 2024 were included in utilization analyses as they contributed pre-period data and could be included in comparisons between not-yet treated and treated units, improving statistical power. In spending analyses, 2024 data were excluded entirely, as no spending data were available for 2024.

*Outcomes*

Inpatient utilization included hospitalizations and observation unit stays. When an ED visit resulted in a connected inpatient encounter, this was considered an inpatient encounter.

*Analyses: Utilization and Spending*

The Callaway and Sant’Anna approach groups treated units by time of treatment, and compares them only to valid comparisons in calendar time, avoiding comparisons between earlier-treated and later-treated units.^4^ At given time points, treated units may be validly compared to: 1) never treated units (i.e., the ineligible comparison group), and 2) not yet treated units. Here, including the not-yet-treated group in comparison decreased baseline differences between ACO- and non-ACO groups, and also increased the sample size of valid comparisons, which was necessary given our small sample.^5^

The aggregate ATT is the preferred effect estimate in studies with a large number of small treated groups where it would be infeasible to evaluate group-level differences based on treatment timing.^6^ The aggregate ATT weights each group’s ATT (groups defined based on treatment timing) by group size.^6^

**Supplemental Table 1.** Summary of MassHealth eligibility criteria for FSP-H

| **Eligibility criteria** | **Definition** |
| --- | --- |
| **ACO member** | Eligible for MassHealth and enrolled with the ACO contractor on: 1) the date FSP-H screening is conducted, 2) the first date of FSP-H service receipt, and 3) every subsequent 90 calendar days from initial FSP-H receipt date until conclusion of episode |
| *and* |  |
| **Health needs based criteria** | Behavioral health need (mental health or substance use disorder) *or* complex physical health need (i.e., persistent, disabling, or progressively life threatening) *or* need for assistance with one or more activities od daily living or instrumental activities of daily living, *or* repeated emergency department use (2 or more visits in 6 months, or 4 or more in a year) *or* experiencing high risk pregnancy *or* complications with pregnancy |
| *and* |  |
| **Experiencing homelessness** | Lacking a fixed, regular, adequate nighttime residence (public or private place, shelter, exiting institution), *or* immediately losing fixed adequate nighttime residence (within 21 days, no plan for subsequent residence), *or,* fleeing domestic violence (and has no other residence or plan) |
| *or* |  |
| **Experiencing risk of homelessness** | Does not have sufficient resources or support networks immediately available to prevent moving to emergency shelter or other place not meant for human habitation *and* has moved because of economic reasons 2+ times in last 50 days, *or* living in home of another person because of economic hardship *or* lives in hotel or motel with cost not covered by charity/government *or* lives in overcrowded unit, *or* has past history of receiving publicly funded services, *or* lives in housing in conditions otherwise associated with instability and risk of homelessness |

Summary adapted from Mass.gov^7^

**Supplemental Table 2. ICD-10 and procedure codes used to identify clinical covariates**

| **Clinical covariate^*^** | **Included conditions** | **Codes** |
| --- | --- | --- |
| Substance use disorder^8^ | Opioid abuse;  Opioid dependence; Opioid use, unspecified; Alcohol abuse; alcohol dependence; alcohol use unspecified; alcoholic polyneuropathy; Alcoholic cardiomyopathy; Alcoholic gastritis without bleeding; Alcoholic gastritis with bleeding; Alcoholic hepatitis without ascites; Alcoholic hepatitis with ascites; Alcohol use complicating pregnancy, unspecified trimester; Alcohol use complicating pregnancy, first trimester; Alcohol use complicating pregnancy, second trimester; Alcohol use complicating pregnancy, third trimester; Alcohol use complicating childbirth; Alcohol use complicating the puerperium; Cannabis abuse; Cannabis dependence; Cocaine abuse; Cocaine dependence; Other stimulant abuse; Other stimulant dependence; Sedative, hypnotic, or anxiolytic abuse; Sedative, hypnotic, or anxiolytic dependence; Hallucinogen abuse; Hallucinogen dependence; Inhalant abuse; Inhalant dependence; Other psychoactive substance abuse; Other psychoactive substance dependence; Drug use complicating pregnancy, unspecified trimester; Drug use complicating pregnancy, first trimester; Drug use complicating pregnancy, second trimester; Drug use complicating pregnancy, third trimester; Drug use complicating childbirth; Drug use complicating the puerperium | *ICD-10:* F111x; F112x, F119x; F1010; F1011; F10120; F10121; F10129; F10130; F10131; F10132; F10139; F1014; F10150; F10151; F10159; F10180; F10181; F10182; F10188; F1019; F1020; F1021; F10220; F10221; F10229; F10230; F10231; F10232; F10239; F1024; F10250; F10251; F10259; F1026; F1027; F10280; F10281; F10282; F10288; F1029; F1094; F10950; F10951; F10959; F1096; F1097; F10980; G621; I426; K2920; K2921; K7010; K7011; O99310; O99311; O99312; F1210; F1211; F12120; F12121; F12122; F12129; F1213; F12150; F12151; F12159; F12180; F12188; F1219; F1220; F1221; F12220; F12221; F12222; F12229; F1223; F12250; F12251; F12259; F12280; F12288; F1229; F1310; F1311; F13120; F13121; F13129; F13130; F13131; F13132; F13139; F1314; F13150; F13151; F13159; F13180; F13181; F13182; F13188; F1319; F1320; F1321; F13220; F13221; F13229; F13230; F13231; F13232; F13239; F1324; F13250; F13251; F13259; F1326; F1327; F13280; F13281; F13282; F13288; F1329; F1410; F1411; F14120; F14121; F14122; F14129; F1413; F1414; F14150; F14151; F14159; F14180; F14181; F14182; F14188; F1419; F1420; F1421; F14220; F14221; F14222; F14229; F1423; F1424; F14250; F14251; F14259; F14280; F14281; F14282; F14288; F1429; F1510; F1511; F15120; F15121; F15122; F15129; F1513; F1514; F15150; F15151; F15159; F15180; F15181; F15182; F15188; F1519; F1520; F1521; F15220; F15221; F15222; F15229; F1523; F1524; F15250; F15251; F15259; F15280; F15281; F15282; F15288; F1529; F1610; F1611; F16120; F16121; F16122; F16129; F1614; F16150; F16151; F16159; F16180; F16183; F16188; F1619; F1620; F1621; F16220; F16221; F16229; F1624; F16250; F16251; F16259; F16280; F16283; F16288; F1629; F1810; F1811; F18120; F18121; F18129; F1814; F18150; F18151; F18159; F1817; F18180; F18188; F1819; F1820; F1821; F18220; F18221; F18229; F1824; F18250; F18251; F18259; F1827; F18280; F18288; F1829; F1910; F1911; F19120; F19121; F19122; F19129; F19130; F19131; F19132; F19139; F1914; F19150; F19151; F19159; F1916; F1917; F19180; F19181; F19182; F19188; F1919; F1920; F1921; F19220; F19221; F19222; F19229; F19230; F19231; F19232; F19239; F1924; F19250; F19251; F19259; F1926; F1927; F19280; F19281; F19282; F19288; F1929; O99320; O99321; O99322; O99323; O99324; O99325 |
| Serious mental illness^8^ | Major depressive disorder, single episode, severe without psychotic features; Major depressive disorder, single episode, severe with psychotic features; Major depressive disorder, recurrent severe without psychotic features; Major depressive disorder, recurrent, severe with psychotic symptoms; bipolar disorder (excluding cyclothymic disorder); Schizophrenia; Delusional disorders; shared psychotic disorder; Schizoaffective disorder, Other psychotic disorder; Unspecified psychosis | *ICD-10:* F320; F321; F322; F323; F324; F325; F329; F330; F331; F332; F333; F3340; F3341; F3342; F339; F3010; F3011; F3012; F3013; F302; F303; F304; F308; F309; F31x; F200; F201; F202; F203; F205; F2081; F2089; F209; F22; F24; F250; F251; F258; F259; F28; F29 |
| Frailty associated with Activities of Daily Living Dependence^9^ | Lipid abnormality; Vertigo; Arthritis and joint conditions; Bladder disfunction; Podiatric care; Heart failure; Psychiatric care; Hypotension or shock; Stroke or brain injury; Dementias; Parkinson’s; Weakness; Skin ulcer; Paralysis; Rehabilitation care; Home oxygen; Ambulance transport; Wheelchair; Home hospital bed; | *ICD-10*: E71.30; E75.21; E75.22; E75.24; E75.3; E75.5; E75.6; E77.0; E78.0; E78.1; E78.2; E78.3; E78.4; E78.5; E78.6; E78.70; E78.79; E78.8; E78.9; E88.1; E88.89; H81.; H82.; H83.0; H83.1; H83.2; R42.; M00.; M01.; M02.1; M02.3; M02.8; M04.2; M04.8; M04.9; M05.; M06.0; M06.1; M06.3; M06.4; M06.8; M06.9; M07.6; M08.0; M08.2; M08.3; M08.4; M08.8; M08.9; M11.1; M11.2; M11.8; M11.9; M12.0; M12.8; M12.9; M13.0; M13.1; M15.; M16.; M17.; M18.; M19.0; M19.1; M19.2; M19.9; M22.0; M22.1; M23.5; M24.0; M24.1; M24.3; M24.4; M24.5; M24.6; M24.7; M24.8; M24.9; M25.0; M25.2; M25.3; M25.4; M25.5; M25.6; M25.9; M32.10; M32.12; M32.13; M32.14; M32.15; M32.19; M32.8; M32.9; M33.; M34.; M35.00; M35.01; M35.02; M35.03; M35.04; M35.09; M35.1; M35.2; M35.3; M35.5; M35.8; M35.9; M36.8; M43.3; M43.4; M43.5X2; M43.5X3; M43.5X4; M43.5X5; M43.5X6; M43.5X7; M43.5X8; M43.5X9; M79.646; N13.; N31.; N32.; N36.; N39.3; N39.4; N39.8; N39.9; R32.; R33.; R39.14; R39.81; L02.61; L03.03; L03.04; L60.; L62.; L84.; I09.81; I11.0; I13.0; I13.2; I25.5; I42.; I43.; I50.; I51.4; I51.5; I51.7; I97.0; I97.11; I97.120; I97.13; I97.19; F01.; F02.; F03.; F04.; F05.; F06.; F07.; F09.; F10.13; F10.14; F10.15; F10.180; F10.182; F10.188; F10.23; F10.24; F10.25; F10.26; F10.27; F10.280; F10.282; F10.93; F10.94; F10.95; F10.96; F10.97; F10.980; F10.982; F11.13; F11.14; F11.15; F11.182; F11.23; F11.24; F11.25; F11.282; F11.93; F11.94; F11.95; F11.982; F12.13; F12.15; F12.180; F12.23; F12.25; F12.280; F12.93; F12.95; F12.980; F13.13; F13.14; F13.15; F13.180; F13.182; F13.23; F13.24; F13.25; F13.26; F13.27; F13.280; F13.282; F13.93; F13.94; F13.95; F13.96; F13.97; F13.980; F13.982; F14.13; F14.14; F14.15; F14.180; F14.182; F14.23; F14.24; F14.25; F14.280; F14.282; F14.93; F14.94; F14.95; F14.980; F14.982; F15.13; F15.14; F15.15; F15.180; F15.182; F15.23; F15.24; F15.25; F15.280; Z51.89; I95.; R57.; R65.2; T79.4; T81.1; G92.; G93.1; G93.4; G93.5; G93.6; G93.89; G93.9; I60.; F06.8; G13.8; G21.0; G25.7; G25.89; G25.9; G26.; G30.; G31.0; G31.1; G31.83; G31.84; G31.85; G31.89; G31.9; G91.1; G91.2; G91.3; G91.8; G91.9; G94.; I69.010; I69.011; I69.014; I69.015; I69.018; I69.019; I69.110; I69.111; I69.114; I69.115; I69.118; I69.119; I69.210; I69.211; I69.215; I69.218; I69.219; I69.310; I69.311; I69.314; I69.315; I69.318; I69.319; I69.810; I69.811; I69.814; I69.815; I69.818; I69.819; I69.91; R41.1; R41.2; R41.3; R41.81; G20.; G21.1; G21.2; G21.3; G21.4; G21.8; G21.9; M62.5; M62.81; M62.84; M62.89; R53.81; R54.; Z74.0; Z74.01; L89.; L97.; L98.4; G81.; G82.; G83.1; G83.2; G83.3; G83.5; G83.89; G83.9; I69.03; I69.04; I69.05; I69.06; I69.13; I69.14; I69.15; I69.16; I69.23; I69.24; I69.25; I69.26; I69.33; I69.34; I69.35; I69.36; I69.83; I69.84; I69.85; I69.86; I69.93; I69.94; I69.95; I69.96; R29.5  *HCPCS/CPT:* 97110; 97161; 97162; 97116; 97535; 92507; 97164; 97012; 97112; 97530; 97113; E1390; E1391; E1392; E0431; E0433; E0434; E0435; E0439; E0441; E0442; E0443; A0426; A0427; A0428; A0429; A0999; E1050; E1060; E1070; E1083; E1084; E1085; E1086; E1087; E1088; E1089; E1090; E1091; E1092; E1093; E1100; E1110; E1140; E1150; E1160; E1161; E1170; K0001; K0002; K0003; K0004; K0005; K0006; K0007; K0008; K0009; E0250; E0251; E0255; E0256; E0260; E0261; E0265; E0266; E0270; E0290; E0291; E0292; E0293; E0294; E0295; E0296; E0297; E0301; E0302; E0303; E0304; E0316 |

^*^Each clinical condition was defined based on presence of at least one ICD-10 or procedure code to maximize sensitivity, and because this aligned with FSP-H risk-based eligibility criteria

**Abbreviations**: **ICD-10**: International Classification of Diseases, 10^th^ edition; **HCPCS**: Healthcare Common Procedure Coding System; **CPT**: Current Procedural Terminology

**Supplemental Table 3. Housing support service types available in FSP-H**

| **Support type** | **Select examples of CMS-allowable supports** |
| --- | --- |
| Pre-tenancy, ongoing coordination individual supports | - Budgeting for living expenses - Applications for tenancy - Obtaining services needed to establish a safe and healthy living environment |
| Pre-tenancy, transitional assistance | - First and last month’s rent - Security deposit - Costs for filing applications |
| Tenancy sustaining, ongoing coordination | - Obtaining tenancy training - Obtaining and maintaining housing benefits and establishing credit |
| Home modifications | - Installation of grab bars and hand showers - Training to use such supplies and modifications correctly |

**Abbreviations: CMS:** Centers for Medicare and Medicaid Services

| **Program characteristic** | **Value** | |
| --- | --- | --- |
| Total individuals engaged, N | 437 | |
| Experiencing homelessness, n (%) | 297 (68) | |
| Experiencing housing instability, n (%) | 140 (32) | |
| Number of services provided, median (IQR) | 2 (1-3) | |
| Days from first to last service, median (IQR) | 62 (29-144) | |
| Total service encounters, N | | 1,148 |
| Pre-tenancy^1^, individual, ongoing coordination, n (%) | | 456 (40) |
| Tenancy-sustaining, ongoing coordination, n (%) | | 314 (27) |
| Pre-tenancy^1^, transitional, household items, n (%) | | 168 (15) |
| Pre-tenancy^1^, transitional, fees and payments, n (%) | | 138 (12) |
| Tenancy-sustaining, household items, n (%) | | 22 (2) |
| Home modification, n (%) | | 25 (2) |
| Pre-tenancy^1^, individual, transportation, n (%) | | 12 (1) |
| Tenancy-sustaining, other^2^, n (%) | | 13 (1) |

**Supplemental Table 4. FSP-H recipients and service delivery, 2021-2024**

**Abbreviations: FSP-H:** MassHealth Flexible Services Program housing supports **IQR:** interquartile range

^1^Pre-tenancy services included services to assist people experiencing homelessness to locate and transition into housing as well as services to help housed or doubled-up recipients move into new housing

^2^Includes fees and payments and transportation; combined to cells with values < 11

**Source**: Data derived from authors’ own analyses of FSP-H, claims, and electronic health records from WellSense and Boston Medical Center Health System.

**Supplemental Table 5. Mean monthly inpatient and ED utilization, and per-patient spending in FSP-H recipients before and after FSP-H, 2021-2024**

| **Month relative to FSP-H start** | **Inpatient utilization rate, mean (95% CI)** | **ED utilization rate, mean (95% CI)** | **Spending, mean (95% CI), $** | **Spending, median (IQR), $** | |
| --- | --- | --- | --- | --- | --- |
| Pre-period |  |  |  | |  |
| 12 months pre | 15.6 (11.5, 19.7) | 14.6 (10.6, 18.6) | 3,734 (2,905, 4,562) | | 857 (216, 3,296) |
| 11 months pre | 14.2 (10.3, 18.1) | 14.6 (10.6, 18.5) | 4,100 (2,830, 5,369) | | 802 (192, 3,454) |
| 10 months pre | 12.7 (9.0, 16.3) | 19.6 (15.2, 24.0) | 3,178 (2,543, 3,813) | | 916 (126, 3,183) |
| 9 months pre | 14.6 (10.7, 18.5) | 14.6 (10.7, 18.5) | 3,511 (2,680, 4,342) | | 754 (103, 3,171) |
| 8 months pre | 14.5 (10.6, 18.3) | 16.6 (12.6, 20.7) | 4,008 (2,831, 5,185) | | 845 (216, 3,750) |
| 7 months pre | 12.2 (8.7, 15.8) | 11.9 (8.4, 15.4) | 3,232 (2,547, 3,917) | | 897 (194, 3,160) |
| 6 months pre | 16.1 (12.1, 20.0) | 15.2 (11.3, 19.0) | 3,737 (2,913, 4,561) | | 870 (163, 3,115) |
| 5 months pre | 12.9 (9.3, 16.5) | 15.9 (11.9, 19.8) | 3,622 (2,883, 4,360) | | 1,029 (199, 3,369) |
| 4 months pre | 16.2 (12.3, 20.1) | 17.1 (13.1, 21.1) | 3,525 (2,789, 4,262) | | 936 (202, 3,283) |
| 3 months pre | 16.8 (12.8, 20.7) | 17.4 (13.3, 21.4) | 4,419 (3,401, 5,436) | | 992 (275, 4,231) |
| 2 months pre | 13.8 (10.2, 17.5) | 15.3 (11.5, 19.1) | 4,476 (3,463, 5,488) | | 1,084 (270, 4,315) |
| 1 month pre | 16.5 (12.5, 20.4) | 17.4 (13.3, 21.4) | 4,090 (3,253, 4,928) | | 897 (282, 4,903) |
| Post-period |  |  |  | |  |
| 0 months post | 14.1 (10.4, 17.8) | 13.5 (9.9, 17.2) | 3,761 (2,885, 4,637) | | 1,023 (310, 3,745) |
| 1 month post | 13.5 (9.9, 17.2) | 13.5 (9.9, 17.2) | 3,404 (2,667, 4,141) | | 978 (273, 3,273) |
| 2 months post | 14.4 (10.7, 18.1) | 14.7 (10.9, 18.5) | 3,123 (2,331, 3,915) | | 841 (282, 3,027) |
| 3 months post | 16.5 (12.5, 20.4) | 12.9 (9.4, 16.5) | 3,621 (2,677, 4,566) | | 1,045 (250, 3,309) |
| 4 months post | 12.5 (8.9, 16.1) | 16.8 (12.7, 20.8) | 2,786 (2,035, 3,537) | | 732 (270, 2,866) |
| 5 months post | 11.4 (7.9, 14.8) | 14.5 (10.6, 18.3) | 2,650 (1,919, 3,381) | | 763 (101, 2,402) |
| 6 months post | 12.9 (9.2, 16.7) | 15.5 (11.5, 19.6) | 2,817 (2,024, 3,610) | | 716 (62, 2,754) |

**Abbreviations**: **FSP-H:** MassHealth Flexible Services Program housing supports; **CI**: confidence interval; **IQR**: interquartile range; **ED**: emergency department

**Source**: Data derived from authors’ own analyses of FSP-H, claims, and electronic health records from WellSense and Boston Medical Center Health System.

| **Month relative to**  **FSP-H start** | **Percentage point difference in monthly inpatient utilization probability, FSP-H vs. untreated (95% CI)** | **Percentage point difference in monthly ED utilization probability, FSP-H vs. untreated (95% CI)** | **Difference in monthly per-patient spending,**  **FSP-H vs. untreated,**  **$ (95% CI)** |
| --- | --- | --- | --- |
| *3+ modified Elixhauser conditions* | | | |
| Pre-period average | 0.8 (-1.0, 2.7) | -0.8 (-2.9, 1.3) | 189 (-86, 464) |
| 12 months pre | 6.4 (-5.2, 18.0) | 1.7 (-12.8, 16.1) | 622 (-938, 2,182) |
| 11 months pre | -4.3 (-16.5, 7.9) | -9.4 (-25.4, 6.6) | -649 (-2,555, 1,257) |
| 10 months pre | -2.0 (-13.0, 9.0,) | 9.8 (-3.7, 23.3) | 80 (-2,088, 2,248) |
| 9 months pre | 10.0 (-2.5, 22.5,) | -13.8 (-27.5, -0.1) | 543 (-1,100, 2,185) |
| 8 months pre | -7.0 (-20.4, 6.5,) | 1.7 (-11.5, 15.0) | 706 (-886, 2,297) |
| 7 months pre | 2.2 (-8.4, 12.8) | -7.7 (-18.0, 2.6) | -452 (-2,043, 1,140) |
| 6 months pre | 7.0 (-4.7, 18.6) | 10.6 (-1.1, 22.2) | 992 (-680, 2,663) |
| 5 months pre | -7.3 (-22.6, 7.9) | -1.0 (-13.3, 11.2) | -725 (-2,353, 903) |
| 4 months pre | 9.4 (-0.7, 19.5) | 4.3 (-7.1, 15.8) | -211 (-1,955, 1,533) |
| 3 months pre | -0.2 (-10.9, 10.5) | -8.1 (-19.8, 3.6) | 1306 (-389, 3,000) |
| 2 months pre | -3.8 (-16.7, 9.0) | -5.2 (-15.4, 5.1) | 966 (-701, 2,632) |
| 1 months pre | -0.3 (-12.2, 11.6) | 7.3 (-4.4, 19.1) | -906 (-2,260, 448) |
| *Substance use disorder* |  |  |  |
| Pre-period average | -0.6 (-9.9, 8.7) | 0.3 (-1.4, 2.0) | 65 (-152, 282) |
| 12 months pre | 3.3 (-7.5, 14.0) | -7.0 (-18.6, 4.5) | 872 (-800, 2,544) |
| 11 months pre | 2.9 (-8.7, 14.5) | 2.3 (-9.1, 13.6) | 612 (-1,290, 2,514) |
| 10 months pre | -3.5 (-12.7, 5.7) | 1.4 (-8.7, 11.4) | -849 (-2,495, 796) |
| 9 months pre | 8.9 (-0.1, 18.0) | -6.7 (-17.1, 3.7) | 111 (-1,168, 1391) |
| 8 months pre | -6.0 (-15.3, 3.3) | 5.7 (-6.9, 18.3) | 748 (-490, 1,985) |
| 7 months pre | -2.4 (-12.5, 7.8) | -6.2 (-17.9, 5.5) | -896 (-2,566, 774) |
| 6 months pre | 6.8 (-3.3 ,17.0) | 4.5 (-6.2, 15.2) | 984 (-559, 2,527) |
| 5 months pre | -5.2 (-15.8, 5.3) | 5.1 (-4.6, 14.9) | -184 (-1,943, 1,576) |
| 4 months pre | 0.1 (-9.9, 10.1) | 3.4 (-7.6, 14.5) | -847 (-2,336, 642) |
| 3 months pre | 0.0 (-12.7, 12.8) | -1.8 (-12.8, 9.1) | 947 (-863, 2,756) |
| 2 months pre | 0.2 (-12.8, 13.2) | -2.0 (-11.3, 7.3) | 320 (-1,549, 2,189) |
| 1 month pre | -12.8 (-124.0, 98.4) | 5.2 (-7.8, 18.2) | -1035 (-2,634, 564) |
| *Serious mental illness* |  |  |  |
| Pre-period average | 0.8 (-1.5, 3.1) | -0.4 (-3.1, 2.2) | 272 (26, 518) |
| 12 months pre | -2.6 (-16.1, 10.9) | -2.4 (-15.3, 10.6) | 721 (-972, 2,414) |
| 11 months pre | 0.9 (-11.7, 13.5) | -7.0 (-18.5, 4.5) | 403 (-1,684, 2,490) |
| 10 months pre | 1.6 (-7.0, 10.1) | 5.2 (-9.5, 19.9) | 666 (-732, 2,063) |
| 9 months pre | -0.5 (-10.7, 9.7) | -10.2 (-23.9, 3.6) | 216 (-1,037, 1,469) |
| 8 months pre | -0.1 (-10.0, 9.8) | -2.2 (-13.8, 9.3) | 43 (-1,146, 1,233) |
| 7 months pre | 0.4 (-8.4, 9.2) | -3.7 (-13.9, 6.5) | 655 (-646, 1,955) |
| 6 months pre | -0.5 (-11.1, 10.0) | 10.1 (-3.7, 23.8) | -213 (-1,500, 1,073) |
| 5 months pre | -1.8 (-14.0, 10.4) | 4.4 (-6.6, 15.4) | -461 (-1,681, 758) |
| 4 months pre | 0.8 (-11.4, 13.0) | -3.6 (-16.8, 9.7) | 11 (-1,304, 1,325) |
| 3 months pre | 4.0 (-5.9, 14.0) | 1.2 (-10.1, 12.5) | 701 (-602, 2,005) |
| 2 months pre | -1.2 (-12.5, 10.0) | -4.1 (-14.6, 6.4) | 1,028 (-529, 2,584) |
| 1 month pre | 8.5 (-1.5, 18.5) | 6.9 (-4.6, 18.4) | -505 (-1,744, 734) |

**Supplemental Table 6. Pre-period trends by subgroup: monthly inpatient utilization, emergency department utilization, and per-patient spending, 2021-2024**

**Abbreviations: FSP-H:** MassHealth Flexible Services Program housing supports; **ED**: emergency department; **CI**: confidence interval

**Supplemental Table 6. Pre-period trends by subgroup: monthly inpatient utilization,**

| **Month relative to FSP-H start** | **Percentage point difference in monthly inpatient utilization probability, FSP-H vs. untreated (95% CI)** | **Percentage point difference in monthly ED utilization probability, FSP-H vs. untreated (95% CI)** | **Difference in monthly spending, FSP-H vs. untreated,**  **$ (95% CI)** |
| --- | --- | --- | --- |
| *Frailty associated with ADL dependence* | | | |
| Pre-period average | 0.9 (-1.0, 2.7) | 1.1 (-0.2, 2.5) | 77 (-118, 273) |
| 12 months pre | 6.1 (-5.7, 18.0) | -3.5 (-12.1, 5.2) | 938 (-533, 2,410) |
| 11 months pre | 1.9 (-6.7, 10.5) | 5.8 (-2.4, 14.0) | 366 (-976, 1,708) |
| 10 months pre | -1.6 (-9.3, 6.0) | 9.4 (0.3, 18.4) | -464 (-1,494, 565) |
| 9 months pre | 0.6 (-7.5, 8.7) | -9.4 (-18.5, -0.4) | -563 (-1,950, 823) |
| 8 months pre | 0.1 (-7.7, 7.9) | 6.0 (-3.1, 15.1) | 887 (-185, 1,959) |
| 7 months pre | 0.6 (-7.3, 8.5) | -7.4 (-16.7, 2.0) | -535 (-1,687, 616) |
| 6 months pre | 4.8 (-3.1, 12.6) | 1.9 (-5.2, 8.9) | 317 (-809, 1,442) |
| 5 months pre | -5.0 (-13.2, 3.2) | 1.5 (-6.2, 9.3) | -457 (-1,512, 597) |
| 4 months pre | 1.9 (-6.0, 9.9) | 8.3 (-0.6, 17.2) | -239 (-1,312, 835) |
| 3 months pre | -1.0 (-10.3, 8.4) | -5.8 (-13.7, 2.1) | 587 (-378, 1,551) |
| 2 months pre | -3.3 (-13.5, 6.9) | -3.0 (-10.8, 4.9) | 525 (-723, 1,774) |
| 1 months pre | 5.4 (-4.3, 15.0) | 9.8 (0.1, 19.5) | -431 (-1,384, 523) |
| *Age 17-54 years* |  |  |  |
| Pre-period average | -0.4 (-1.4, 0.6) | 0.2 (-0.8, 1.1) | 56 (-91, 203) |
| 12 months pre | 3.0 (-4.8, 10.9) | -2.0 (-9.9, 5.9) | 756 (-505, 2,017) |
| 11 months pre | -4.3 (-12.1, 3.5) | -4.1 (-12.6, 4.4) | -267 (-1,506, 972) |
| 10 months pre | 2.7 (-4.5, 9.9) | 8.9 (-0.1, 17.9) | -182 (-1,316, 952) |
| 9 months pre | 0.2 (-7.1, 7.5) | -8.0 (-16.4, 0.4) | -460 (-1,482, 562) |
| 8 months pre | -2.0 (-9.5, 5.5) | 3.0 (-5.7, 11.7) | 572 (-399, 1,544) |
| 7 months pre | -3.6 (-10.8, 3.5) | -4.0 (-12.4, 4.3) | 204 (-868, 1,276) |
| 6 months pre | 4.2 (-3.1, 11.5) | 4.7 (-4.3, 13.7) | 49 (-1,061, 1,159) |
| 5 months pre | -8.2 (-15.9, -0.6) | 2.4 (-5.5, 10.3) | -69 (-1,274, 1,135) |
| 4 months pre | 2.4 (-4.8, 9.6) | 3.2 (-4.2, 10.7) | -135 (-894, 623) |
| 3 months pre | 5.5 (-2.2, 13.1) | -3.5 (-11.5, 4.5) | 603 (-389, 1,595) |
| 2 months pre | -3.8 (-10.8, 3.1) | 0.3 (-7.2, 7.9) | -166 (-1,381, 1,048) |
| 1 month pre | -0.9 (-9.1, 7.3) | 1.0 (-7.4, 9.3) | -231 (-1,251, 789) |
| *Age 55+ years* |  |  |  |
| Pre-period average | -0.7 (-3.0, 1.7) | 1.8 (-1.2, 4.7) | 246 (-95, 588) |
| 12 months pre | -0.9 (-12.5, 10.7) | 1.6 (-8.9, 12.2) | 526 (-1,326, 2,377) |
| 11 months pre | -0.4 (-12.1, 11.4) | 3.4 (-12.5, 19.3) | 440 (-1,540, 2,420) |
| 10 months pre | -4.5 (-16.0, 6.9) | 8.3 (-6.6, 23.2) | -143 (-1,956, 1,670) |
| 9 months pre | 2.5 (-8.7, 13.6) | -15.5 (-29.2, -1.7) | -205 (-1,913, 1,503) |
| 8 months pre | -2.9 (-15.9, 10.2) | 2.4 (-9.7, 14.6) | -383 (-1,674, 909) |
| 7 months pre | -1.3 (-11.8, 9.3) | -4.2 (-16.7, 8.3) | 44 (-1,328, 1,415) |
| 6 months pre | 7.2 (-5.0, 19.5) | -0.1 (-9.8, 9.6) | 500 (-1,197, 2,197) |
| 5 months pre | 6.3 (-12.2, 24.8) | 9.2 (-8.4, 26.8) | -68 (-1,921, 1,786) |
| 4 months pre | -0.4 (-14.2, 13.4) | -1.8 (-18.6, 15.0) | 417 (-1,551, 2,385) |
| 3 months pre | 2.5 (-20.3, 25.2) | 5.1 (-13.5, 23.9) | 338 (-1,738, 2,415) |
| 2 months pre | -1.9 (-19.4, 15.6) | -1.1 (-16.3, 14.1) | 982 (-1,394, 3,359) |
| 1 month pre | -14.2 (-35.0, 6.6) | 13.7 (-4.5, 31.9) | 505 (-2,046, 3,056) |

**emergency department utilization, and per-patient spending, 2021-2024, cont.**

**Abbreviations: FSP-H:** MassHealth Flexible Services Program housing supports; **ED**: emergency department; **CI**: confidence interval; **ADL:** activities of daily living

**Source**: Data derived from authors’ own analyses of FSP-H, claims, and electronic health records from WellSense and Boston Medical Center Health System.

**Supplemental Table 7. Staggered difference-in-difference sensitivity analyses**

| **Sensitivity analysis specification** | **Inpatient utilization** | | **ED utilization** | | | **Spending, $** | | |
| --- | --- | --- | --- | --- | --- | --- | --- | --- |
|  | *ATT coefficient*  *(95% CI)* | *P-value* | *ATT coefficient*  *(95% CI)* | | *P-value* | *ATT coefficient*  *(95% CI)* | *P-value* | |
| Unadjusted OLS model, base-case outcome definitions^1,2^ | -3.3  (-8.1, 1.6) | 0.188 | -3.1  (-7.8, 1.5) | 0.191 | | -524  (-1,081, 33) | | 0.065 |
| *Outcome definition variations* |  |  |  | | |  | | |
| Inpatient utilization: change in monthly visit count | -0.08  (-0.22, 0.05) | 0.236 | NA | | | NA | | |
| ED utilization: change in monthly visit count | NA | | -0.04  (-0.18, 0.11) | 0.600 | | NA | | |
| Monthly per-patient spending, no top-coding | NA | | NA | | | -505  (-1,197, 188) | | 0.153 |

**Abbreviations: ATT:** average treatment effect on the treated; **CI:** confidence interval; **ED:** emergency department; **OLS:** ordinary least squares

^1^Base-case outcome definitions were: monthly rate of inpatient and ED utilization (coded as a binary probability per month) and monthly spending, top-coded at the sample’s 99^th^ percentile ($32,000)

^2^Supplemental Figures 1 and 2 and show event study coefficients for the unadjusted OLS model specification to assess pre- and post-trends unconditional on covariates

**Source**: Data derived from authors’ own analyses of FSP-H, claims, and electronic health records from WellSense and Boston Medical Center Health System.

**Supplemental Figure 1. Difference-in-differences event study plot, unadjusted ordinary least squares (OLS) model: utilization outcomes in FSP-H vs. unexposed comparators, 2021-2024**

**Abbreviations: FSP-H:** MassHealth Flexible Services Program housing supports

**Legend**. Difference-in-differences event study plots for inpatient (A) and emergency department (B) utilization. Plots show the average treatment effect on the treated (ATT) between FSP-H and unexposed comparison groups, in each month relative to FSP-H start. Blue points represent pre-period months, and orange points represent post-period months. Pre-period and post-period averages are shown. Results are shown for an unadjusted, ordinary least squares regression model. Note that pre- and post- averages differ from the aggregate ATT (in Supplemental Table 6), as they are simple averages across time and not weighted by group size.

**Source**: Data derived from authors’ own analyses of FSP-H, claims, and electronic health records from WellSense and Boston Medical Center Health System.

**Supplemental Figure 2. Difference-in-differences event study plot, unadjusted ordinary least squares (OLS) model: per-patient monthly spending in FSP-H vs. unexposed comparators, 2021-2024**

**Abbreviations: FSP-H:** MassHealth Flexible Services Program housing supports

**Legend**. Difference-in-differences event study plot for per-patient monthly spending. Monthly spending is capped at the sample’s 99^th^ percentile. The plot shows the average treatment effect on the treated (ATT) between FSP-H and unexposed comparison groups, in each month relative to FSP-H start. Blue points represent pre-period months, and orange points represent post-period months. Pre-period and post-period averages are shown. Results are shown for an unadjusted, ordinary least squares regression model. Note that pre- and post- averages differ from the aggregate ATT (in Supplemental Table 6), as they are simple averages across time and not weighted by group size.

**Source**: Data derived from authors’ own analyses of FSP-H, claims, and electronic health records from WellSense and Boston Medical Center Health System.

**Supplemental Endnotes**

1.	Mass.gov. Boston Accountable Care Organization in Partnership with BMC HealthNet Plan. Accessed June 3, 2025. https://www.mass.gov/info-details/boston-accountable-care-organization-in-partnership-with-bmc-healthnet-plan

2.	McCann NC, Ettinger de Cuba S, Hofman M, et al. Validation of an Electronic Health Record Algorithm for Identifying Housing-Related Needs in a Safety-Net Health System. *Health Services Research*. Published online In Press.

3.	Kim J, Wang H, Ma J, et al. Addressing Social Determinants of Health in Primary Care Patients May Reduce Hospital Readmissions: A Quasi-Experimental Study. *J Gen Intern Med*. 2024;39(16):3104-3112. doi:10.1007/s11606-024-08813-8

4.	Callaway B, Sant’Anna PHC. Difference-in-Differences with Multiple Time Periods. *Journal of Econometrics*. 2021;225(2):200-230. doi:10.1016/j.jeconom.2020.12.001

5.	Rios-Avila F, Callaway B, Sant’Anna PHC. csdid: Difference-in-Differences with Multiple Time Periods in Stata. Stata Conference. 2021. Accessed October 31, 2025. https://www.stata.com/meeting/us21/slides/US21_SantAnna.pdf

6.	Sant’Anna PHC, Callaway B, Quispe A, Guevara C. CSDID: Introduction. 2024. Accessed November 7, 2025. https://d2cml-ai.github.io/csdid/examples/csdid_basic.html

7.	Appendix M- Flexible Services Program Enrollee Eligibility and Allowable Services. Mass.gov. n.d. Accessed January 26, 2024. https://www.mass.gov/doc/pcaco-appendix-m/download

8.	Larochelle MR, Lodi S, Yan S, Clothier BA, Goldsmith ES, Bohnert ASB. Comparative Effectiveness of Opioid Tapering or Abrupt Discontinuation vs No Dosage Change for Opioid Overdose or Suicide for Patients Receiving Stable Long-term Opioid Therapy. *JAMA Netw Open*. 2022;5(8):e2226523. doi:10.1001/jamanetworkopen.2022.26523

9.	Duchesneau ED, Shmuel S, Faurot KR, et al. Translation of a Claims-Based Frailty Index From the *International Classification of Diseases, Ninth Revision, Clinical Modification* to the Tenth Revision. *American Journal of Epidemiology*. 2023;192(12):2085-2093. doi:10.1093/aje/kwad151
